# Where next for structural bioinformatics?

**DOI:** 10.1371/journal.pbio.3003903

**Published:** 2026-07-27

**Authors:** Joe G. Greener

**Affiliations:** Medical Research Council Laboratory of Molecular Biology, Cambridge, United Kingdom

## Abstract

Structural bioinformatics aims to answer biological questions by considering biomolecular structures at scale. This Perspective argues that now that we have accurate predictions, we need to ask what we can learn from them.

## Teaser

Structural bioinformatics aims to answer biological questions by considering biomolecular structures at scale. We now have accurate predictions and need to ask what we can learn from them.

I still remember the day I found out about AlphaFold 2 [1]. Sitting at home in November 2020, shortly before the virtual critical assessment of protein structure prediction (CASP)14 conference, where protein structure prediction methods are assessed on unseen targets, an email pinged in. In typical CASP fashion, this table of results was not a simple ranking of each group’s performance; it was a large file that required processing. No matter how many times I tried to sort it, it seemed like one group had smashed it across the board. Then I realized that my script was fine, the problem had effectively been solved. My first thought was excitement. My second thought was, where does this leave structural bioinformatics?

Despite a few predictions of its decline, structural biology is in rude health. Getting the structure is only part of the process, and AlphaFold only works some of the time. You get the structure in order to interpret it and learn things, often with experiments, so AlphaFold has given structural biology a boost and has become an important part of the toolkit. However, true single sequence structure prediction has remained elusive. The cautious old guard who initially responded to AlphaFold by highlighting the difference between protein structure prediction (the what) and protein folding (the how), correctly saying that the second remains an open problem, has surprised nobody by not going on to work on protein folding.

CASP has struggled for funding, been given a reprieve by Google, and continues to assess the many AlphaFold variants. Companies have been drawn in, assembling and then sometimes laying off biological research groups. Structural bioinformatics has evolved somewhat. Is this a good thing? Well, let us consider the main aims of the discipline. I exclude molecular dynamics, which is more focused on physics than data, and sequence-based tasks such as sequence searching, which do not tend to use structure even if the sequences are of proteins.

The first main aim is to understand biomolecules by considering structures at scale, rather than individually. This is how the field began, when the Protein Data Bank started getting big enough to reveal patterns in evolution and function. Structural classification databases such as CATH and SCOP were a natural consequence of this.

The second main aim is to make predictive models, with the idea that biologists would use these on their system of interest. This includes prediction of structure, function, interactions, binding sites, and other tasks such as structural alignment. Associated with this aim is producing databases of predictions using these models. On a deeper level, this is partly an attempt to understand the world, since you do not understand what you cannot predict. As we will come on to, though, being able to predict something does not necessarily mean that you do understand it.

And, although debatable as to whether it falls under the banner of structural bioinformatics, a third aim is the design of new biomolecules. Given that modern protein design and structure prediction use similar models, it seems fair to include it and, more broadly, the design of drugs and organisms.

How are we doing with respect to those aims? Structural bioinformatics in 2026 seems to mainly consist of three things: using AlphaFold, using large language models (LLMs), and protein design.

First, what I like to call ‘AlphaFoldology’: reproducing, retraining, explaining, ablating, using for screening, tuning multiple sequence alignment inputs, estimating errors, extending to ensembles, integrating experimental data. A hot topic is whether and how AlphaFold can output different conformations of fold-switching proteins [2,3]. This might help with the study of individual systems, but I struggle to get enthusiastic about the fundamental question. Does it teach us something about the world if a black box model predicts one thing or another? AlphaFoldology is directly concerned with the second and third aims above and does little to increase our scientific understanding on its own. Nonetheless, I find myself excited about the release of OpenFold and the associated ablations that will let us peek inside the crystal ball. Recent announcements of the Structural Genomics Consortium Target 2035 and OpenBind efforts to increase the amount of protein–ligand data are welcome, and point to a better understanding of drug binding as well as the obvious outcome of more data for training predictive models.

Second, LLMs are being trained on protein, and increasingly DNA, sequences. These have found wide use as foundation models that ‘compress biology’ and work well as inputs for other supervised models (second aim) and to generate sequences (third aim). Whether they can produce genuinely new things or are just efficient samplers is an ongoing research area [4], and sadly, one where you have to deal with the bluster of multiple startups. ESM3 can supposedly simulate “500 million years of evolution” [5], which is an interesting way of saying that they designed a fluorescent protein with 58% sequence identity to a known fluorescent protein. Again, LLMs infiltrating every corner of bioinformatics does not seem to lead to understanding directly, and may hinder it by reducing the interpretability of inputs for downstream models. Until we can formulate our questions in a closed, maths Olympiad style that is suitable for modern reasoning models, it is not clear that we can extract the biological reasoning that we are after.

Third, advances are being made in protein design, mainly of binders but increasingly of enzymes and proteins with specific desirable features. This has seen an explosion of work and venture funding in the past few years, driven by the rise of generative artificial intelligence in general and the repurposing of AlphaFold-like architectures in particular. This is a clear success story with widely available computational methods, even if experimental success has a high barrier of entry. Models like RFdiffusion get a lot of the attention, but ProteinMPNN [6] deserves much of the credit for making inverse folding routine. This is clearly associated with the third aim, although we can learn principles from it too [7].

The theme is that there is a lot of prediction and design, and not much focus on what we can learn with all this [8]. We now have the AlphaFold database, foundation models that supposedly span all scales of biology, and we seem to be able to design binders to arbitrary proteins. Should we not have tried to use these to learn something? I do not mean for individual systems, biologists do that, I mean we should use the data deluge to do some informatics.

There have, of course, been efforts to cluster the AlphaFold database [9,10], and while these do provide insights themselves, the main output seems to be to provide processed data for others to use. The Encyclopedia of Domains (TED) takes a careful, domain-based approach and provides more CATH annotations [11]. This treasure trove of data can surely be used for more in the future. We spent so long trying to get all the protein structures, we should have thought more about what we were going to do with them once we had them.

How about looking at evolution through the lens of structure and comparing proteomes across the tree of life, which will tell us when evolution introduced new protein folds to carry out a function rather than reusing existing ones? Or understanding the link between structure and function, without cheating by transferring annotations from homologs, since so many sequences with a function annotation now have a structure available? Or explaining the origin and function of unusual protein chemistries [12], where a handful of examples may have become thousands? What about unsupervised protein classification and comparison to human classifications to reveal our biases? Or trying to find the unifying principles of nebulous concepts like allostery, since we can find conserved pockets distant from the active site and analyze residue contact graphs at scale? Or wading into the liquid–liquid phase separation debate? Or even working out how much of a simplification Anfinsen’s dogma, that structure is solely determined by sequence, is [13]?

I know there are people doing these things, yet I cannot help but feel that structural bioinformatics should move beyond making predictions and start to ask what those predictions can teach us. We cannot leave all the fun to the biologists; we should zoom out while they zoom in. We should, please, not let the field become a load of half-baked LLMs.
